# Role of Endourological Procedures (PCNL and URS) on Renal Function: a Systematic Review

**DOI:** 10.1007/s11934-020-00973-4

**Published:** 2020-04-21

**Authors:** Thomas Reeves, Amelia Pietropaolo, Nariman Gadzhiev, Christian Seitz, Bhaskar K. Somani

**Affiliations:** 1 Department of Urology, University Hospital Southampton NHS Foundation Trust, Tremona Road, Southampton, SO16 6YD UK; 2 Department of Urology, Pavlov First Saint Petersburg State Medical University, Lva Tolstogo 17, Saint-Petersburg, Russian Federation 197342; 3 Department of Urology, Comprehensive Cancer Centre, Medical University of Vienna, Vienna, Austria

**Keywords:** Renal function, eGFR, Ureteroscopy, PCNL, Creatinine, Chronic kidney disease

## Abstract

**Purpose of Review:**

To present the latest evidence related to the impact of ureteroscopy (URS) and percutaneous nephrolithotomy (PCNL) on the renal function.

**Recent Findings:**

Our review suggests that the overall renal function is not detrimentally affected by endourological interventions (URS, PCNL). This is however influenced by the preoperative renal function, presence of comorbidities such as diabetes and hypertension. For PCNL procedures, tract multiplicity, preoperative UTI, and postoperative bleeding also contribute to a decline in renal function.

**Summary:**

This review suggests that endourological interventions do not adversely affect renal function and tend to improve it in patients who do not have a poor renal function prior to the procedure. Several factors including poor preoperative renal function, diabetes, hypertension, and multiple percutaneous tracts appear to predispose patients to declining renal function after procedure, and these patients should be counseled for and followed up appropriately.

## Introduction

Kidney stone disease (KSD) is rising with a lifetime prevalence of 14% [1–3]. Surgical options such as shockwave lithotripsy (SWL), percutaneous nephrolithotomy (PCNL), and ureteroscopy (URS) are all used as treatment modalities [4–6]. The chosen treatment often depends on stone characteristics, patient fitness, comorbidities, surgical expertise, and underlying renal function. Preoperative assessment of these patients involves up-to-date imaging, urine culture, renal function, and fitness for a general anesthetic. The overall incidence of KSD has been rising, and hence more patients are subjected to surgical intervention [7, 8].

Kidney function can be impaired as a result of the disease, urinary infections, or ureteric obstruction related to the stone or surgical intervention related to the KSD. While it is generally believed that treatment of KSD would lead to an improvement of renal function, it is unclear if the surgical procedure required to remove the stone will have an adverse effect. There is a theoretical risk of deterioration of renal function with both PCNL and URS. The physical puncturing of the kidney during PCNL causes direct damage to the renal parenchyma, and this is amplified as PCNL is increasingly used to treat complex or staghorn calculi requiring multiple puncture tracts [9]. During endoscopic approach to the urinary tract, high pressure irrigation is often required to maintain a visual field, causing dilatation of the renal calyces that could potentially harm the function of the kidney. In addition, while the renal parenchyma is not breached as with PCNL, application of the holmium:yttrium-aluminum-garnet (Ho:YAG) laser may cause heat related tissue damage [10, 11].

Given the theoretical risk of renal function decline with both PCNL and URS, our aim was to conduct systematic review to clarify the effect of endourological interventions on renal function.

## Method

### Search Strategy

Our systematic review was performed as per the Cochrane guidelines and Preferred Reporting Items for Systematic Reviews and Meta-analyses (PRISMA) checklist [12]. The databases searched included MEDLINE, Cumulative Index to Nursing and Allied Health Literature (CINAHL), Excerpta Medica Database (EMBASE), Scopus, Clinicaltrials.gov, Google Scholar, Cochrane library and Web of Science with references cross-checked, and individual urology journals also hand-searched. The search terms included “stones,” “calculi,” “urolithiasis,” “nephrolithiasis,” “kidney,” “renal,” “ureteroscopy,” “URS,” “laser,” “fragmentation,” “percutaneous,” “PCNL,” “mini,” “miniaturized,” “percutaneous nephrolithotomy,” “lithotripsy,” “renal function,” “kidney function,” “chronic kidney disease,” “CKD,” “creatinine,” “eGFR,” “MAG3,”and “DMSA.” The references of identified studies were examined to find any further potential studies for inclusion. Boolean operators (AND, OR) were employed. The research was limited to English language articles from 1990 to June 2019.

A cut off of ten patients was set to include studies from centers with minimum relevant endourological experience in managing stones. All original studies were included, and where more than one article was available, the study with the longest follow-up was included. Experienced reviewers (TR, AP) not involved in the original work independently identified all the studies that appeared to fit the inclusion criteria, which were then included for a full review. All discrepancies were resolved with mutual agreement and consensus with the senior author (BKS).

### Inclusion Criteria

1. Studies reporting on renal function of patients following endourological intervention (PCNL and URS)
2. Studies reporting on a minimum of 10 patients
3. Studies available in English

### Exclusion Criteria

1. Laboratory, animal data, or review articles
2. Studies published before 2000

### Data Extraction and Analysis

The following variables were extracted from included studies: author, year of publications, journal, country of study, treatment modality, patient characteristics, stone characteristics, method of monitoring renal function, follow up, and pre- and postoperative renal function. Data were collected using Microsoft Excel 2019 (version 16.28). Due to the heterogeneity of the included studies, the authors decided that meta-analysis of effect sizes was not suitable, and hence either pooled analysis was performed to calculate mean values or outcomes were summarized in a narrative fashion.

### Quality of Studies Assessment

The Centre for Evidence-Based Medicine criteria were used to evaluate the levels of evidence of the included studies [13]. The quality of reporting outcomes was performed according to the Strengthening the Reporting of Observational Studies in Epidemiology (STROBE) statement [14].

## Results

### Study Selection and Characteristics

**Fig. 1:**
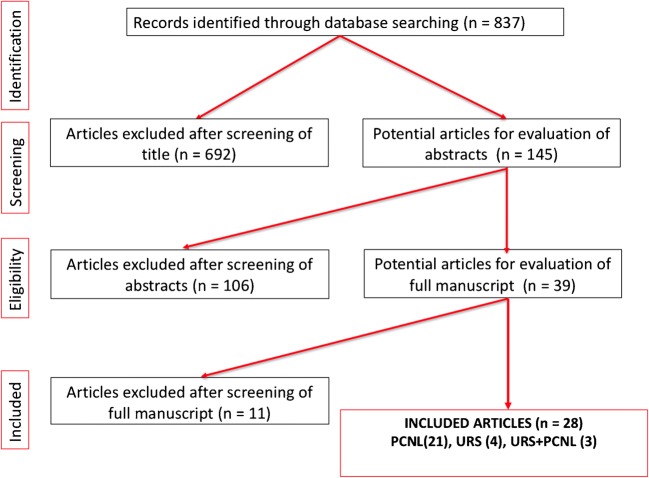
PRISMA flow chart of the included studies

The literature search yielded 837 publications (Fig. 1). After excluding reports that were out of the scope of our systematic review, 145 abstracts were reviewed of which 39 full articles were reviewed for inclusion. Twenty-eight studies were included in the final review (5 were excluded as they were published before 1990, 4 did not mention the effect on renal function, 1 was an animal study and 1 was not in English language). Included studies were published between 2001 and 2019. Three papers compared the effect of PCNL and URS on renal function (Fig. 1).

### PCNL

The effect of PCNL on renal function was assessed in 21 studies published between 1999 and 2019. This included 1994 patients, and the mean age of patients was 49.3 years (Tables 1 and 2). The follow-up in these studies ranged from 1 day to 51 months [15, 16]. While 11 studies [15–26] used blood test to measure renal function, 1 study [27] used radionucleotide scans and 8 studies used combination of both [28–35].

**Table 1:** Study details

	Author	Year	Journal	Country	Level of evidence	N	Age (years)	male/female	SFR	Mean operation time (min)	Average stone size (mm)	Cumulative stone diameter (mm2)	Diameter of the largest stone(mm)	Number of stones	Location	Composition
PCNL	Chatham	2001	Adult urology	USA	II	45	49 (11–75)	6//13	68%	2.57 h (1.17–5.08)	38	1432 (156–5220)	NS	NS	NS	26% calc ox/calc phos, 21% calc ox, 16% struvite, 11% calc phos, 11% UA, 5% cystine, 5% matrix, 5% not-analyzed
	Singh	2001	International urology and nephrology	India	III	70	41.43 (9–70)	68//22	75%	NS	NS	928.6	NS	NS	NS	Mixed, calc ox mono, calc ox di, and struvite stones were encountered in 48%, 14%, 17% and 21% respectively
	Kukreja	2003	Journal endourology	India	III	84	47 ± 14	64//20	85.70%	NS		1564 ± 1568	NS	Staghorn 35	35 staghorn, 22 pelvic, 7 caliceal, 32 complex	NS
	Al-Kohlany	2005	Journal of urology	Egypt	I	43	NS	17//26	74%	127 ± 30	187 ± 69	Complete staghorn	NS	NS	NS	UA stones in 14 (28%), calc ox mono in 12 (24%), mixed calc ox and UA in 6 (12%), struvite in 5 (10%), cystine in 2 (4%), and mixed stones In 11 (22%)
	Handa	2006	Journal endourology	USA	III	196	NS	NS	NS	NS	NS	NS	NS	NS	NS	NS
	Hegarty	2006	Journal endourology	USA	III	20	54.4 ± 12.4	15//5	100%	NS	NS	2157 ± 1441 (single tract); 423 ± 299 (multi tract)	NS	staghorn	NS	NS
	Moskovitz	2006	Journal endourology	Israel	II	88	47 ± 16	47//41	90.00%	NS	> 150	NS	NS	staghorn	NS	NS
	Yaycioglu	2007	Urological research	Turkey	III	38	59 ± 16	13//6	72.7% IRF;84.2% NRF	175 ± 69	54 ± 20 (IRF); 42 ± 24 (NRF)	NS	NS	staghorn	NS	IRF; calc ox 10, UA 4, Mg Am Phos 2, UA & calc ox 2, calc ox/phos 1. NRF calc ox 12, UA 2, UA/calc ox 2, and, Mg Am Phos 1
	Kilic	2008	Journal endourology	Turkey	II	24	36.67 ± 14.68	12//12	NS	122 ± 43	68.5 ± 32.7	NS	NS	NS	NS	NS
	Kuzgunbay	2010	Journal endourology	Turkey	II	16	NS	NS	75.00%	NS	NS	Complete staghorn	NS	NS	NS	calc ox 9, UA 2, UA and calc 2, Mg Am 2, calc phos and oxalate 1
	Unsal	2010	Journal endourology	Turkey	II	50	43.6	37//13	92%%	80.5 (40–170)	NS	NS	NS	NS	NS	NS
	Akman	2012	Journal of urology	Turkey	III	265	44.4 ± 15.3	NS	76.50%	80 ± 35	NS	1191 ± 732	NS	staghorn	NS	calc ox in 44, MG Am phos 13, UA 13, cystine 3, calc ox and phos 5, UA& calc phos in 2, Mg Am phos & calc ox 2
	Chen	2012	Urology	China	I	273	49.2 (22–73)	165//108	84.70%	129.2 ± 27	55 ± 7	NS	NS	staghorn	NS	NS
	Ozden	2012	Urology	Turkey	III	67	57 ± 14.1	47//20	76.80%	88.5 ± 5.7	NS	670 ± 65	NS	staghorn 11	Pelvis 13, Pelvis plus polar 28, Polar 14, Staghorn 11, Upper ureter 3,	3 UA, 16 infection, 24 calc ox, 4 calc phos
	Fayad	2014	Journal endourology	Egypt	II	102	39.9 (35–76)	70//32	70.70%	NS	NS	NS	NS	NS	NS	NS
	El Tabey SK	2014	Urology	Egypt	III	200	52.3 ± 11.7	133/67	89.50%	NS	NS	NS	NS	Multiple 39, staghorn 66, single 95	NS	NS
	Pérez-Fentes	2014	Urolithiasis	Spain	II	30	60.1 ± 13.1	9//21	73%	94 (69,134)	NS	356 (220–820)	NS	NS	NS	NS
	EL-Nahas	2016	BJUI	Egypt	I	42	50 ± 11	16//54	60%	US-130 ± 34; L-148 ± 35	NS	L–15985 ± 8320; US–16656 ± 8211	NS	staghorn	NS	NS
	Gorbachinsky	2016	Journal of urology	USA	II	110	53 ± 15.4	52//58	NS	NS	NS	NS	NS	NS	NS	NS
	Piao*	2016	World journal urology	South Korea	II	31	58.0 ± 13.2	20//11	85.10%	88.6 (±66.9)	NS	3754 ± 6757	19.1 ± 13.2	2.9 ± 2.7	NS	calc ox mono 81 (54.7%) calc ox di 29 (19.6%) UA29 (19.6%) Carbonate apatite 9 (6.1%)
	Zhou	2017	Journal endourology	China	III	178	53.7 ± 15.0	78//100	NS	NS	783 ± 605	NS	NS	NS	NS	NS
	Shi	2018	BJUI	China	III	53	47.34 ± 12.24	26//27	92.45%	117.55 ± 49	50 ± 24.77	NS	NS	Staghorn 17, Multiple 32	MP 2, LP1, pelvis 1, staghorn 17, multiple 32	NS
	Jiao*	2019	Medicine	China	III	58	51.67 ± 12.29	39//19	87.93%	90.40 ± 31.29	12.27 ± 4.39	NS	NS	NS	Renal pelvis or prox ureter 7; UC/MC 25; LC 26	NS
	Cho*	2019	Scientific reports	Korea	II	20	57.3 ± 13.5	14//6	85.50%	72.1 ± 57.0	17.2 ± 13.5	3345 ± 7115	17.2 ± 13.6	3.1 ± 3.2	NS	calc ox mono 64 (54.7); calc ox dehydrate 20 (17.1); UA 25 (21.4); Carbonate apatite 8 (6.8)
URS	Ingimarsson	2012	Urology	USA	II	113	56	55//58	91.40%	NS	7 ± 4	905	NS	NS	Bilateral renal or ureteropelvic junction stones without ureteral stones 71 (60.7%), renal and ureteral 21.4% Bilateral renal stones with unilateral ureteral stones 25 (21.4%) Bilateral renal stones with bilateral ureteral stones 2 (1.7%)	calc ox 83 (71%); apatite 21 (18%); brushite 6 (5%); UAin 5 (4%); AmU in 2 (2%)
	Sninsky	2014	Journal of Endourology	USA	III	26	54.3 ± 14.5	12//14	NS	NS	25 ± 12	NS	NS	NS	45% kidney; 55% kidney & ureter	NS
	Hoarau	2015	IBJU	France	III	163	52.8 ± 17	86//77	74.40%	96.4 ± 40.78	15 ± 9	NS	12.9 ± 5.7	NS	NS	calc ox mono 34%, calc ox dehydrate 5%, UA 8%, carbapatite 16.6%, and unknown 53%
	Yang B	2016	Urologia Internationalis	China	III	44	46.3 ± 10.1	29//15	86.40%	94.8 ± 29.0	26 ± 6	NS	NS	3.0 ± 0.8	Pelvis (14.6%) UC (11.5%) MC (1.69%) LC 47.7%) Ureter (9.2%)	NS
	Piao*	2016	World Journal Urology	South Korea	II	117	58.0 ± 13.2	74//43	85.10%	88.6 ± 66.9	NS	3754 ± 6757	19.1 ± 13.2	2.9 ± 2.7	NS	calc ox mono 81 (54.7%) calc ox di 29 (19.6%) UA29 (19.6%) Carbonate apatite 9 (6.1%)
	Jiao*	2019	Medicine	China	III	48	55.69 ± 12.70	33//15	81.25%	105.56 ± 45.76	12 ± 4	NS	NS	NS	Pelvis or proximal ureter 8; UC/MC 11: LC 29	NS
	Choo*	2019	Scientific reports	Korea	II	97	57.3 ± 13.5	66//31	85.50%	72.1 ± 57.0	NS	3345 ± 7115	17.2 ± 13.6	3.1 ± 3.2	NS	calc ox mono 64 (54.7); calc ox di (17.1); UA 25 (21.4); Carbonate apatite 8 (6.8)

*Studies listed twice as include both URS and PCNL; *IRF* impaired renal function; *NRF* normal renal function; *US* ultrasound group; *L* laser group; *Calc* calcium; *ox* oxalate; *UA* uric acid; *phos* phosphate; *Mg* magnesium; *Am* ammonium; *mono* monohydrate; *di* dihydrate; *NS* not specified

**Table 2:** Effect of PCNL and URS on the renal function of patients

	Author	Year	Measure	Mean follow up time	Mean preoperative renal function	Postoperative change	Study summary
PCNL	Chatham	2001	creatinine, radionucleotide scan	15 months	0.9 mg/dL	No significant change. Trend to improvement	PCNL does not result in loss of renal function
	Singh	2001	eGFR, radionucleotide scan	9 months	NS	No significant change. Trend to improvement	The average fall in serum creatinine values was 1.53 mg/dl (32%) and the average functional improvement by renal dynamic scans stood at 20.7%
	Kukreja	2003	Creatinine	2.2 ± 1.34 years	2.87 ± 1.19 mg/dL	No significant change	33 patients (39.3%) showed improvement, 24 (28.6%) showed stabilization, and 27 (32.1%) showed deterioration in renal function. Poor pre-operative function linked to deterioration.
	Al-Kohlany	2005	eGFR, creatinine, radionucleotide scan	4.8 ± 2.5 months	1 ± 0.3 mg/dL	No significant change. Trend to improvement	Split GFR of the treated kidneys improved or remained stable in 91% patients
	Handa	2006	Creatinine	1 day	0.97 ± 0.02 mg/dL	Significantly worse	Significant increase in serum creatinine of 0.14 mg/dL at 1 day
	Hegarty	2006	Creatinine	NS	1.67 ± 1.33 mg/dL	No significant change in single-tract group. Significantly worse in multiple-tract group	Significant rise in serum creatinine (1.67 mg/dL to 1.91 mg/dL; *P* ± 0.05) and drop in creatinine clearance (76.9 mL/min to 67.2 mL/min; P ± 0.05) in the multiple-tract group; this was more pronounced in patients with existing renal insufficiency. No significant change in renal function was seen in the single-tract group
	Moskovitz	2006	Radionucleotide scan	15–24 months	NS	No significant change	No significant change
	Yaycioglu	2007	Creatinine	15.6 months	2.8 mg/dL	No significant change. Trend to improvement	IRF group mean serum creatinine value was 2.8 mg/dl before surgery and 2.6 mg/dl after. NRF Group mean serum creatinine levels before and after were 0.93 ± 0.16 and 0.94 ± 0.17 mg/dl.
	Kilic	2008	Creatinine	12 months	0.83 ± 0.42 mg/dL	No significant change	PCNL does not cause obvious renal dysfunction and significant parenchymal scarring
	Kuzgunbay	2010	Creatinine	51.1. ±10/1	2.30 ± 0.56 mg/dL	No significant change. Trend to improvement	Creatinine values decreased to normal range in six patients (37.5%), six patients (37.5%) had stable renal function and values increased in four patients (25%)
	Unsal	2010	Creatinine, radionucleotide scan	6 months	1.19 ± 0.46 mg/dL	No significant change	QSPECT of 99mTc-DMSA confirms that renal function is preserved or often improved after percutaneous stone removal
	Akman	2012	eGFR, creatinine	36. ±24. months	1.08 ± 0.51 mg/dL	No significant change. Trend to improvement	Renal function was improved or maintained in 80% patients
	Chen	2012	eGFR, creatinine, radionucleotide scan	6 months	0.94 ± 25.4 mg/dL	Significant improvement	A combination of multitract MPCNL and high-power Ho:YAG laser does not delay postoperative renal function recovery
	Ozden	2012	eGFR	45.7 ± 17.08 months	37.9 ± 14.05 ml/min	No significant change. Trend to improvement	Mean eGFR was preoperatively 37.9 (±14) and postoperatively 45.1 (±16). Diabetes mellitus and urinary infection were predictive of renal function deterioration at 1 year on multivariate analysis
	El Tabey	2014	eGFR	3 ± 1.4 years	2 ± 0.8 mg/dL	Significant improvement	Significant improvement in renal function at long term follow-up
	Fayad	2014	creatinine, radionucleotide scan	12 months	1.52 ± 0.56 mg/dL	Poor preoperative function significantly worsened. Normal function no significant change	Patients with normal preoperative renal function showed no significant change, those with baseline renal impairment showed significant worsening. Risk factors for this were elevated (1.4 mg/dL) preoperative serum creatinine level, diabetes, and hypertension
	Pérez-Fentes	2014	eGFR, creatinine, radionucleotide scan	3 months	74.73 ± 24.5 ml/min; 1.0 ± 0.4 mg/dL	No significant change	PCNL has a minimal impact on global kidney function. Perioperative complications increased PCNL functional damage
	EL-Nahas	2016	eGFR	3 months	0.9 ± 0.04 mg/dL	No significant change	4 patients improved, 2 decreased, 36 stationary renal function
	Gorbachinsky	2016	Creatinine, radionucleotide scan	4.1 months	1.08 ± 0.49 mg/dL	No significant change. Trend to worse in multi tract group	2.28% decrease in function in multi tract group
	Piao*	2016	eGFR, creatinine, radionucleotide scan	3 months	78.3 ± 26.2 ml/min, 1.1 ± 0.4 mg/dL	No significant change. Trend to improvement	Abnormal separate renal function showed postoperative recovery in 31 patients (58.5%), three cases (5.7%) showed deterioration
	Zhou	2017	eGFR, radionucleotide scan	7.6 (6–12) months	29.8 ± 21.2 ml/min	Significant improvement. No significant difference in single/multiple tracts	Significant improvement. No significant difference in single/multiple tracts
	Shi	2018	eGFR	6 months	1.15 ± 0.48 mg/dL	No significant change. Trend to improvement	Mean eGFR was 78.58 post op compared withn75.51 (27.08) pre op. Diabetes and high pre-operative creatinine predictive of postoperative decline
	Cho*	2019	eGFR, creatinine	60–90 days	0.99 ± 0.31 mg/dL	Poor preoperative function significantly worsened. Normal function no significant change	Preoperative severe deterioration of separate renal function was a significant predictor for the postoperative deterioration of renal function. Low preoperative deterioration showed high probability of recovery
	Jiao*	2019	eGFR	1 month	87.45 ± 49.73 ml/min	No significant change	Mean change in eGFR 87.45- > 89.21 difference not statistically significant
URS	Ingimarsson	2012	Creatinine	2.8 years	0.99 mg/dL	No significant change. Trend to improvement	Mean creatinine was 0.99 (SD 0.28) preoperatively and 1.00 (SD 0.29) postoperatively
	Sninsky	2014	eGFR	28.1 months (5–75)	68 ± 13.3 ml/min	No significant change. Trend to improvement	The mean eGFR improved from 68.0 - > 75.4, not statistically significant
	Hoarau	2015	eGFR	15.5 ± 11.5 months	84.30 ± 26.2 ml/min	No significant change. Trend to improvement	Significant renal function deterioration occurred in 8 cases (4.9%) and significant renal function amelioration occurred in 23 cases. Median GFR was not significantly changed from 84.3 ± 26.2 to 84.9 ± 24.5
	Piao*	2016	eGFR, creatinine, radionucleotide scan	3 months	78.3 ± 26.2 ml/min; 1.1 ± 0.4 mg/dL	No significant change. Trend to improvement	Abnormal separate renal function showed postoperative recovery in 31 patients (58.5%), 3 cases (5.7%) showed deterioration
	Yang B	2016	Creatinine	4 weeks	1.40 ± 1.68 mg/dL	Significant improvement	Mean serum creatinine improved from 81– > 75
	Choo*	2019	eGFR, radionucleotide scan	60–90 days	NS	Poor preoperative function significantly worsened. Normal function no significant change	Preoperative severe deterioration of separate renal function was a significant predictor for the postoperative deterioration of renal function. Low preoperative deterioration showed high probability of recovery
	Jiao*	2019	eGFR	1 month	74.46 ± 17.50 ml/min	No significant change. Trend to improvement	Mean eGFR 74.46- > 77.83 not statistically significant

NS not specified

Three studies showed significantly improved renal function following PCNL [19, 30, 35]. Eight studies showed no significant improvement but a trend toward improved renal function [16, 17, 23–25, 28, 29, 34]. Eight studies showed no significant change in renal function [18, 21–23, 26, 27, 32, 34]. Handa et al. showed that on day 1 post-procedure the renal function was significantly worse [15].

Hegarty et al. showed significantly worse renal function in patients who underwent multiple tracts PCNLs, but no significant change in those with single tract approach [20]. Fayad et al. showed that those with poor preoperative renal function had significantly worsened renal function post-procedure, but those with normal preoperative function had a stable renal function [31]. Fayad, Ozden, and Chi et al. showed that diabetes was associated with poor postoperative renal function [17, 23, 31]. In addition, Fayad et al. and Ozden et al. showed that postoperative UTI was associated with poor postoperative renal function [17, 31]. Perez-Fentes et al. suggested that postoperative complications were associated with more parenchymal damage following PCNL [33].

### Ureteroscopy

The effect of ureteroscopy on renal function was assessed in four studies published between 2014 and 2019 [36–39] (Tables 1 and 2). This included 608 patients, 355 males and 253 females, and the mean age of patients was 54.9 years (Table 1). The follow-up ranged from 4 weeks to 28.1 months [36, 40]. All 4 studies used blood tests (creatinine, eGFR) for renal function monitoring [36–39].

Yang et al. [36] showed that URS significantly improve postoperative renal function. The other three studies showed no statistically significant change but trend to improvement in postoperative renal function [37–39].

## Comparative Studies between PCNL and URS

Three studies included both PCNL and URS published between 2016 and 2019 (Tables 1 and 2). This included 262 patients with a mean age of 57.3 (Table 1) [40–42]. The follow-up ranged from 60 days to 90 days. Jiao et al. and Cho et al. used blood tests (creatinine, eGFR) to measure renal function while Piao et al. used combination of blood test and radionucleotide scans [40–42].

Both Piao et al. and Jiao et al. showed no significant change in renal function but a trend towards improvement [40, 42]. Cho et al. showed that if preoperative renal function was normal and then postoperative renal function was statistically normal, but if the renal function was abnormal, then it had a tendency to deteriorate significantly postoperatively [41].

## Quality Assessment

The Centre for Evidence-Based Medicine criteria were used to evaluate the levels of evidence of the included studies and found that 3 studies were level one [18, 28, 30], 11 were level two [16, 21, 26, 27, 29, 31–33, 38, 41, 42], and 14 were level three evidence ( [15, 17, 19, 20, 22–25, 34–37, 39, 40])(Table 1). In addition, the quality of all studies was assessed for inclusion against the STROBE criteria [14].

## Discussion

### Meaning of the Study

Here we present the only systematic review on the effect of PCNL and URS on renal function. Our study suggests that overall renal function is not detrimentally affected by endourological intervention, but there are potentially some important predictive factors including preoperative renal function, diabetes, and hypertension, hence patients should be appropriately counseled and followed up.

For patients undergoing PCNL, the results were varied. Handa et al. showed a significantly worse postoperative renal function but their follow up time frame was only 1 day, and this may not have been replicated at subsequent follow up [15]. Gorbachinsky et al., Hegarty et al., and El-Tabey et al. showed that multiple tracts were predictive of significant deterioration in renal function [19, 20, 32]. This is perhaps a reflection of the theoretical risk of parenchymal damage causing a decline in renal function, but this wasn’t replicated across all studies using multiple tracts. Several studies showed that a poor preoperative renal function was predictive of the postoperative function [23, 31, 41]. Additionally, Fayad et al. showed that diabetes and hypertension were independent risk factors for poor outcome [31], El-Tabey et al. showed that postoperative bleeding was a factor [19], and Ozden et al. showed that diabetes and urinary tract infection were independent factors [23]. This suggests that declining renal function maybe attributable to patient comorbidities and other underlying disease as opposed to the effect of the endourological procedure alone. Especially as three studies showed significant improvement in function, and the majority of others showed a trend toward improvement [19, 30, 35].

With patients undergoing ureteroscopy only, Yang et al. showed a significant improvement in postoperative renal function [36]. Cho et al. demonstrated that poor preoperative renal function predicated deterioration, but the renal function was protected for those with good pre-operative renal function [41]. All the other studies showed a trend towards improvement of renal function. Interestingly Sninksy et al. concluded that there was no association between poor preoperative function or multiple procedures on the post-procedural function [37].

### Strengths, Limitations, and Areas for Future Research

This study gives and overview of the effect of endourological techniques effect on renal function. Due to the heterogeneity of the studies and methods for monitoring renal function meta-analysis was not possible; this also made it difficult to compare the studies directly. The patient population inherently contains a number of confounders in terms of comorbidities. In addition, many of papers were retrospective case series and prone to bias. It is prudent that future studies look at the procedural cost differences and quality of life in these patients [43–45]. Similarly, the laser settings and the heat generated by them need to be addressed especially in the context of patients with poor-preoperative renal function [46].

The review highlights that although the renal function is unaffected in most endourological interventions, yet there is a lack of prospective real-life data addressing this issue. Similarly, perhaps there is a need for a randomized control trial addressing both PCNL and URS, with an emphasis on pre- and postoperative renal function, taking into consideration the comorbidities such as diabetes, hypertension, obesity, and chronic kidney disease [47]. This is especially important as previous studies have shown a direct link of these factors on the renal function [48]. Identification of high-risk patients and periodic monitoring of renal function would help in early intervention and is likely to protect further deterioration [49]. PCNL does not seem to result in loss of renal function [29]. However, increasing multiplicity of tracts seems to negatively impact the renal function [50]. Minimally invasive PCNL however does not seem to effect renal function even when there are multiple tracts [35]. In patients with pre-existing CKD or diabetes/hypertension and non-obstructed pelvicalyceal system multi-tract PCNL may result in a kidney function deterioration and thus endoscopic combined intrarenal surgery (ECIRS) should be contemplated [51].

## Conclusion

This review suggests that endourological interventions do not adversely affect renal function and tend to improve it in patients who do not have a poor renal function prior to the procedure. Several factors including poor preoperative renal function, diabetes, hypertension, and multiple percutaneous tracts appear to predispose patients to declining renal function after procedure, and these patients should be counseled for and followed up appropriately.
